# Traditional Chinese medicine in the prevention of diabetes mellitus and cardiovascular complications: mechanisms and therapeutic approaches

**DOI:** 10.3389/fphar.2025.1511701

**Published:** 2025-04-11

**Authors:** Caixia Chen, Hui Gao, Ying Wei, Yaxi Wang

**Affiliations:** ^1^ Inner Mongolia Key Laboratory of Medical Cell Biology, Clinical Medicine Research Center, Affiliated Hospital of Inner Mongolia Medical University, Hohhot, Inner Mongolia, China; ^2^ College of Life Sciences, Inner Mongolia Agricultural University, Hohhot, Inner Mongolia, China; ^3^ Thoracic Surgery Department, Inner Mongolia Hospital of Peking University Cancer Hospital, The Affiliated Cancer Hospital of Inner Mongolia Medical University, Hohhot, Inner Mongolia, China; ^4^ Ultrasonic Department, Affiliated Hospital of Inner Mongolia Medical University, Hohhot, Inner Mongolia, China

**Keywords:** traditional Chinese medicine (TCM), diabetes mellitus and cardiovascular complications (DACC), TCM compound prescriptions, TCM botanical drugs, TCM monomers

## Abstract

Diabetes mellitus (DM) is a chronic endocrine and metabolic disorder characterized by persistent hyperglycemia that poses serious threats to human health and quality of life. The morbidity, disability, and mortality rates of cardiovascular complications stemming from chronic hyperglycemia are primary factors affecting the lifespan of patients with diabetes. Currently, there is no cure for DM. Standard biomedical treatments mostly control the symptoms using insulin injections or oral hypoglycemic drugs. Although the effect of standard biomedical therapy is remarkable, its long-term use is prone to toxic side effects. Numerous studies have recently found that Traditional Chinese Medicine (TCM) has strong advantages in the prevention and treatment of DM and cardiovascular complications (DACC). The collection, processing, preparation and clinical use of TCM are guided by the theory of TCM and follow the “holistic concept.” Multiple components, pathways, and targets form the basis for the use of TCM in treating multiple parts and organs of the body simultaneously. TCM is mainly derived from natural medicines and their processed products and has fewer side effects. TCM is clinically used as compound prescriptions, botanical drugs, and monomers. TCM, either independently or in combination with standard biomedical treatments, has shown unique therapeutic advantages. This review aimed to explore the recently reported mechanisms of action of TCM in the prevention and treatment of DACC. These findings will aid the optimization of the current therapy or formation of a therapeutic schedule for integrated TCM and standard biomedical treatments.

## 1 Introduction

Diabetes mellitus (DM) encompasses a group of clinical syndromes caused by genetic, environmental, and other factors, primarily characterized by hyperglycemia. This condition arises from insufficient insulin secretion and/or impaired insulin action, caused by glucose, lipid, and protein metabolism disorders. Diabetes can be categorized into type 1 diabetes mellitus (T1DM) and type 2 diabetes mellitus (T2DM), based on differences in pathogenesis, symptoms, complications, and treatment strategies (Table 1). With increasing population aging and lifestyle changes, the prevalence of DM is increasing dramatically worldwide (Diabetesatlas, 2021). Based on projections, the number of individuals with DM will increase to 642.8 million by 2030 and 783.7 million by 2045 (Collaborators, 2023). Patients with DM face a higher risk of developing cardiovascular disease compared with those without DM, and they often experience these complications earlier in life (Heather et al., 2022). In addition, extensive cohort observational studies conducted over the last 30 years have confirmed that 49% of DM-related deaths are due to cardiovascular disease (Chiquette and Chilton, 2002; Tamayo et al., 2023). Patients with T2DM exhibited a considerably increased lifetime risk of various cardiovascular conditions, which may include coronary artery disease, stroke, heart failure, atrial fibrillation, and peripheral artery disease (Marx et al., 2023). The rising morbidity associated with DM, coupled with cardiovascular complications resulting from long-term hyperglycemia, leads to disability and mortality among patients. This situation has become a pressing public health issue, posing a serious threat to human health (Lopez-Diez et al., 2021).

**TABLE 1:** List of diabetes classification and its conditions.

Subtype	Cause	Affected population	Symptoms	Complications	Treatment method
T1DM	Damage to pancreatic islet function and insufficient secretion of pancreatic β cells	Mostly in adolescence to youth	Onset is acute, with polydipsia, polyphagia, polyuria, weight loss, and emaciation	Diabetic ketoacidosis, hyperosmolar coma, diabetic nephropathy	Insulin injection
T2DM	Caused by multiple factors leading to insufficient insulin secretion or insulin resistance in the body	Mostly in middle - aged people, with a high incidence in people over 40 years old	Onset is slow, without obvious symptoms, and more common in obese people	Diabetic macrovascular disease	Oral hypoglycemic drugs

Cardiomyocytes and vascular endothelial cells are important structural components of the heart and blood vessels. The mechanisms by which long-term hyperglycemia induces cardiomyopathy in DM include at least three key aspects: 1) hyperglycemia-induced oxidative stress promotes the excessive release of reactive oxygen species, which in turn induces cardiomyocyte apoptosis, 2) the oxidative stress may also result in persistent inflammatory damage to blood vessels, thereby increasing the risk of diabetic cardiomyopathy, and 3) hyperglycemia adversely affects protein structure. Studies have shown that hyperglycemia can alter protein structures, leading to the cross-linking of collagen molecules. This alteration impairs the degradation ability of collagen, resulting in increased myocardial fibrosis and infarction (Dal Canto et al., 2023; Stultz and Edelman, 2003). The mechanisms underlying DM-induced chronic hyperglycemia and its associated cardiovascular complications are complex. First, the normal vascular endothelium serves as the body’s natural physical barrier, which can weaken the adhesion of substances such as leukocytes and platelets. However, when endothelial cells are damaged, adhesion molecules become highly expressed, and chemokines are secreted, promoting the adhesion, rolling, and infiltration of leukocytes and platelets into the intimal layer, thus inducing atherosclerosis (Li Y. et al., 2023). Second, high levels of blood glucose are catalyzed by aldose reductase and other enzymes, resulting in the production of fructose and sorbitol, which accumulate outside the endothelial cells. This increase in extracellular osmotic pressure disrupts the internal environment, leading to endothelial cell degeneration, edema, and vascular diseases (Katakami, 2018). In addition, high blood glucose levels damage the inhibitory effect of the renin-angiotensin system, promoting sodium retention, causing insulin resistance (IR), and resulting in hyperinsulinemia. These changes activate the sympathetic nervous system, contributing to hypertension (Jia and Sowers, 2021). Studies have found that the incidence of hypertension in patients with DM is approximately two to six times higher than in those without DM (Lithovius et al., 2020; Wang B. et al., 2022). Chronic hyperglycemia in DM results in a decrease in insulin efficiency in promoting glucose uptake and utilization, alongside a reduction in insulin sensitivity, also known as IR. IR can decrease endothelium-dependent vasodilation response (Fan et al., 2024). The role of vascular endothelium in regulating blood vessel movement is impaired when vascular endothelial dysfunction occurs. Consequently, blood vessel wall elasticity decreases and plaques gradually form on the vessel walls, exacerbating conditions such as atherosclerosis, hypertension, myocardial infarction, stroke, and congestive heart failure. Furthermore, DM is a recognized risk factor for coronary artery disease and is a leading cause of cardiovascular disease and mortality among patients (Association, 2019; Baena-Diez et al., 2016). Statistically, the mortality rate from coronary heart disease in patients with T2DM is two to four times higher than that in patients without DM (Benjamin et al., 2019). In terms of treatment, compared with patients with non-DM-related cardiovascular complications, patients with DM-related cardiovascular complications are less likely to opt for surgical interventions. This is primarily because of the limitations posed by anesthesia evaluation and surgical contraindications. Therefore, exploring and identifying suitable drugs for the prevention and treatment of DM and cardiovascular complications (DACC) is essential.

Currently, several drugs are used to treat DACC. For example, statins, antilipids, and antiplatelet drugs (such as aspirin and clopidogrel) can delay further narrowing of the coronary arteries. When angina symptoms arise from coronary ischemia, nitrates can be used to dilate the blood vessels and improve symptoms. However, each of these medications has various side effects, which may exacerbate long-term complications associated with chronic conditions such as DACC (Table 2). Metformin is one of the most commonly prescribed medications for DM globally. Studies have shown that metformin causes serious gastrointestinal side effects (Dixon et al., 2023). Statins used to treat DACC are associated with an increased risk of developing T2DM (Laakso and Fernandes Silva, 2023). Table 2 outlines the side effects of other commonly used medications. Therefore, it is crucial to identify effective drugs, have fewer side effects, and can contribute to optimizing treatment plans.

**TABLE 2:** Adverse reactions or side effects of commonly used drugs for diabetes and its cardiovascular complications.

Category	Representative drug names	Main indications	Mechanism	Main adverse reactions
Insulin	Insulin injection	T1DM and T2DM, Diabetic ketoacidosis, Gestational diabetes, Hyperkalemia	Exogenous supplementation of insulin	Hypoglycemia
Sulfonylurea secretagogues	Glimepiride	Newly diagnosed, obese type 2 diabetes; after dietary control, fasting or postprandial blood glucose is still high	Stimulate insulin secretion by pancreatic β cells	Hypoglycemia, gastrointestinal discomfort, allergies, etc.
Biguanides	Metformin	T1DM and T2DM	Promote the uptake of glucose by peripheral tissues such as muscles, and inhibit glucose neogenesis; inhibit or delay the absorption of glucose in the gastrointestinal tract	Gastrointestinal reactions, manifested as loss of appetite, diarrhea, metallic taste in the mouth, or fatigue, weight loss, etc.
α - Glucosidase inhibitors	Acarbose	T2DM	Inhibit α - glucosidase on the intestinal mucosa, slowing down the speed of starch decomposition into glucose, reducing and delaying the absorption of glucose in the small intestine	Abdominal distension, abdominal pain, diarrhea, nausea, vomiting, and may also have gastrointestinal spastic pain, intractable constipation, etc.
Thiazolidinediones	Pioglitazone	T2DM	Insulin sensitizer	Liver damage, weight gain, edema, induced hypoglycemia, etc.
Meglitinides	Repaglinide	T2DM	Insulin secretagogue	Hypoglycemia, obesity, elevated transaminases, gastrointestinal reactions, etc.
GLP - 1 inhibitors	Semaglutide	T2DM	Increase insulin secretion, inhibit gastric acid secretion, slow down gastric emptying and suppress appetite	Hypoglycemia, gastrointestinal discomfort, dizziness, fatigue, nausea, diarrhea, vomiting, constipation, abdominal pain, dyspepsia, anorexia, etc.
DPP4 inhibitors	Sitagliptin	T2DM	Promote the release of insulin by pancreatic β cells, and also inhibit pancreatic α cells to avoid excessive secretion of glucagon, increasing insulin levels in the body	Headache, hypoglycemia, nasopharyngitis, upper respiratory tract infection, rash, diarrhea, cough, etc.
Sodium - glucose cotransporter 2 (SGLT2) inhibitors	Empagliflozin	T2DM	Inhibit the reabsorption of glucose by the kidneys	Urinary and genital tract infections, hypoglycemia, diabetic ketoacidosis, etc.
Diuretics	Furosemide Hydrochlorothiazide	Heart failure, hypertension	Reduce circulating blood volume through its diuretic and natriuretic effects, and reduce vascular tension by reducing the sodium content in the vascular wall	Cardiac toxicity, electrolyte imbalance, hypokalemia, hyponatremia, frequent urination
β - receptor blockers	Betaxolol	Hypertension, myocardial ischemia, arrhythmia	Selectively bind to β - adrenergic receptors, thereby antagonizing the stimulating effect of neurotransmitters and catecholamines on β receptors	Cause fatigue, hypotension, bradycardia, breathing difficulties, etc.
Calcium channel blockers	Nifedipine	Hypertension, coronary heart disease, and arrhythmia	Block the calcium channels on the cell membranes of myocardial and vascular smooth muscle cells, inhibit the influx of extracellular calcium ions into the cells, and reduce the intracellular calcium ion level	Hypotension, dizziness, palpitations, constipation, etc.
Angiotensin - converting enzyme inhibitors (ACEI)	Captopril	Hypertension, congestive heart failure and myocardial infarction, diabetic nephropathy and other nephropathies	Inhibit the activity of angiotensin, thereby Reducing the production of angiotensin II, resulting in vasodilation, reduced blood volume, and decreased blood pressure	Dry cough, angioedema, hypotension, renal function impairment, hyperkalemia, etc.
Angiotensin II receptor antagonists (ARB)	Irbesartan	Hypertension	Selectively block the angiotensin II receptor (AT1 type), blocking the effects of angiotensin II (Ang II) to constrict blood vessels, raise blood pressure, promote aldosterone secretion, fluid and sodium retention, and sympathetic nerve excitation	Dizziness, hypotension, hyperkalemia, etc.
Aminocyl heart glycosides	Digoxin	Heart failure, arrhythmia	Interfere with the sodium - potassium pump in myocardial cells, increase the intracellular calcium ion concentration, and improve the contractility of the myocardium, thereby increasing the cardiac output	Arrhythmia, visual changes, nausea, diarrhea, etc.
Anticoagulants	Warfarin	Various thrombi	Antithrombotic formation, antiplatelet aggregation, antifibrinolytic, inhibition of platelet adhesion, inhibition of coagulation factor activity	Bleeding, aggravation of hereditary hemorrhagic diseases, liver function damage, osteoporosis, allergic reactions, etc.
Antiplatelet drugs	Aspirin	Prevention of the formation of blood clots after transient ischemic attack, myocardial infarction, artificial heart valve and venous fistula or other surgeries	Inhibit the growth of cyclooxygenase in platelets	Gastrointestinal bleeding, thrombocytopenia
Lipid - lowering drugs	Statins	Lowering blood lipids, inhibiting the formation of atherosclerosis and thrombosis, and also having various effects such as alleviating the rejection reaction after organ transplantation, treating osteoporosis, anti-tumor, and anti-Alzheimer’s disease	Inhibit the synthesis and release of cholesterol and triglycerides	Muscle pain, abnormal liver function, skin discomfort, gastrointestinal symptoms, liver and kidney function damage

Traditional Chinese medicine (TCM) has gained popularity as a treatment method for chronic diseases in recent years. TCM typically includes TCM compound prescriptions, botanical drugs, and individual botanical drugs components. Compared with standard biomedical treatments, patients benefit from TCM’s “holistic concept,” as well as the characteristics of natural products and lower toxicity. Numerous basic studies have explored TCM’s efficacy in treating DACC (Liu et al., 2023; Song Z. et al., 2023; Zhu et al., 2022). Studies have confirmed that TCM can be used as a complementary treatment method for patients with DM and coronary heart disease (Wei Y. et al., 2022). Although high-quality clinical studies on TCM for the treatment of DACC remain limited, some key studies have provided valuable and compelling evidence for its application. For instance, the MUST-D study, a randomized, double-blind, placebo-controlled phase IV trial, enrolled 716 patients with DM-related coronary heart disease from 97 tertiary hospitals in China (Zhou J. et al., 2023). The study found that the treatment group with Shexiang Baoxin pills was highly effective in reducing the incidence of major adverse cardiovascular events (MACE). Compared with the control group, the incidence of MACEs in the Shexiang Baoxin pill treatment group decreased by 45.8%. Moreover, at 24 months, the overall incidence of secondary endpoints (composite outcome of all-cause death, nonfatal myocardial infarction, nonfatal stroke, hospitalization due to unstable angina or heart failure, and coronary angioplasty) significantly decreased by 32.3%. In addition, the incidence of cardiovascular adverse events in the Shexiang Baoxin pill group (4.4%) was lower than that in the placebo group (7.7%), demonstrating its safety. TCM has also shown remarkable potential in pre-DM intervention. The FOCUS randomized clinical trial included 885 patients with impaired glucose tolerance, abdominal obesity, or any abnormal index of metabolic syndrome (Ji et al., 2024). The results revealed that the risk of DM in the Jinlida Granule group significantly decreased by 41% and had prominent advantages in improving multiple metabolic indices (waist circumference, body mass index, blood glucose, blood lipids, and insulin resistance index). These results reveal the potential of TCM in pre-DM intervention and the improvement of metabolic disorders, providing solid evidence for the further application of TCM for DM prevention and treatment. Furthermore, regarding the treatment of DM complicated by coronary heart disease, a treatment regimen combining Tongxinluo, Jinlida, and metoprolol also presents promising application prospects. One study indicated that the total effective rate of the combined treatment group was significantly higher than that of the single metoprolol treatment group and surpassed the control group in terms of improving cardiac function indices (such as cardiac stroke volume and left ventricular ejection fraction) and blood glucose control (Yujun et al., 2020). Numerous small-sample clinical trials have demonstrated that TCM plays a crucial role in the treatment of DACC (Lili and Yan, 2025; Zhaolong, 2025). For instance, in a study investigating the impact of Danhu Tongbi Decoction on patients with T2DM complicated by coronary heart disease and angina pectoris, the control group received conventional treatment. In contrast, the observation group received Danhu Tongbi Decoction. The results showed that the observation group showed more significant improvements in glycometabolic and lipid-metabolic indices (including blood glucose and lipid levels). Furthermore, there was a notable enhancement in cardiac function, as measured by the left ventricular ejection fraction, and both the frequency and severity of angina attacks were better alleviated (Lili and Yan, 2025). Another study evaluating the efficacy of naoxintong in treating diabetes mellitus complicated by stroke classified patients into control and treatment groups. In addition to the conventional treatment, the treatment group received naoxintong. After a period of treatment, this group showed more substantial improvements in the neurological deficit score, better blood glucose control, and an increase in the daily activity of living ability scores (Zhaolong, 2025). A large number of small-sample clinical studies are sufficient to confirm the therapeutic value of TCM. These findings established a solid foundation for the promotion and communication of TCM in the international DACC field. To reduce the side effects of standard biomedical treatments and increase their efficacy, some studies have used a combination of TCM and standard biomedical treatments. Wang et al. (Wang H. et al., 2024) selected Jinghong Decoction combined with metformin sustained-release tablets for the syndrome differentiation treatment of T2DM. They found that patients’ symptoms improved significantly and adverse reactions were reduced. In summary, by consolidating research progress on TCM compound prescriptions, botanical drugs, and individual monomers aimed at treating DACC in recent years, we aimed to contribute to the development of new effective prevention and treatment strategies.

## 2 The application of TCM compound prescriptions in DACC

TCM compound prescriptions show the characteristics of multiple targets and mechanisms in the treatment of diseases owing to their complex components. In this study, we summarized the mechanisms through which TCM compound prescriptions function in DACC, including reducing inflammation, regulating immunity, modulating gut microbiota, inhibiting islet cell or cardiovascular cell apoptosis, anti-oxidative stress, regulating glucose and lipid metabolism, and other mechanisms (Table 3).

**TABLE 3:** Lists of TCM compounds with potential anti-diabetes and its cardiovascular complications action.

TCM compounds	Composition	Diseases	Model	Optimal dose	Duration of treatment	Effects	Potential mechanism	References
Gegen Qinlian decoction	*Coptis* Salisb, *Scutellaria baicalensis* Georgi, *Pueraria lobata* (Willd.) Ohwi, *Anemarrhena asphodeloides* Bunge, Panax quinquefolius L.,*Paeonia lactiflora* Pall., and *Zingiber officinale* Roscoe	T2DM	GK rats and Wistar rats	22 g/kg	12 weeks	Lowered glucose	①②	Xu et al. (2020)
Baihu Renshen decoction	*Anemarrhena asphodeloides* Bunge, Gypsum, Glycyrrhiza Tourn. ex L., *Oryza sativa subsp. japonica* Shig.Kato, and *Panax ginseng* C.A.Mey	T2DM	T2DM	One dose per time, two times per day	4 weeks	Decreased the levels of pro-inflammatory cytokines and inhibited the oxidative stress, altered the diversity of gut microbiotra	①②	Yao et al. (2022)
Shen-Qi Compound Formula	*Panax ginseng* C.A. Mey., *Astragalus membranaceus Fisch. ex Bunge Fisch. ex Bunge Fisch. ex Bunge* Fisch. ex Bunge, *Salvia miltiorrhiza* Bunge, *Rheum palmatum* L., *Trichosanthes kirilowii* Maxim., *Rehmannia glutinosa* (Gaertn.) Libosch. ex DC., and *Cornus officinalis* Sieb. et Zucc.	T2DM	Wistar rats	1.44 g/kg	8 weeks	Reduced the blood glucose fluctuations, decreased the level of serum LPS, increased the concentrations of SIgA and ZO-1 in intestinal tissue, inhibited the intestinal inflammatory injury and reduce the tissue damage index (TDI)scores	①②	Zhang et al. (2024a)
Jinlida granules	*Panax ginseng* C.A. Mey., Polygonatum odoratum (Mill.) Druce, Atractylodes lancea (Thunb.) DC.,*Sophora flavescens* Aiton, Liriope Herb., *Rehmannia glutinosa* (Gaertn.) *Libosch*. ex DC., *Polygonum multiflorum* Thunb., *Cornus officinalis* Siebold & Zucc., *Poria cocos* (Schw.) Wolf, *Eupatorium fortunei* Turcz., *Coptis chinensis* Franch., *Anemarrhena asphodeloides* Bunge, *Epimedium* Tourn. ex L., *Salvia miltiorrhiza* Bunge, *Pueraria thomsonii* Benth., *Litchi chinensis* Sonn., Lycium chinense Mill.	T2DM	Not mentioned	Not mentioned	Not mentioned	Antidiabetes	①③⑥	Gu et al. (2024)
Danggui Sini San	*Angelica sinensis* (Oliv.) Diels, *Cinnamomum cassia* Presl, *Paeonia lactiflora* Pall., *Atractylodes macrocephala* Koidz., *Bupleurum chinense* DC., *Citrus aurantium* L., *Tetrapanax papyrifer* (Hook.) K. Koch, *Glycyrrhiza uralensis* Fisch, *Astragalus membranaceus* Fisch. ex Bunge, and *Pueraria lobata* (Willd.) Ohwi	T2DM	Patients	Take 400 mL twice a day orally	3 months	Lowered blood sugar and protected cardiovascular function	①③	Wu (2024)
Fufang Zhenzhu Tiaozhi capsule	*Coptis chinensis* Franch., *Ligustrum lucidum* W.T.Aiton, *Salviae Miltiorrhizae* Radix et Rhizoma, *Cirsium japonicum* Fisch. ex DC., *Eucommia ulmoides* Oliv., *Citrus medica L. var. sarcodactylis* (Hoola van Nooten) Swingle, *Panax notoginseng* (Burk.) F.H. Chen, and *Atractylodes macrocephala* Koidz	DM-related coronary heart disease	Chinese Wuzhishan minipigs	1.2 g/kg	22 weeks	Ameliorated coronary atherosclerosis in diabetes mellitus-related coronary heart disease	①③⑥	Wang et al. (2022b)
			HUVECs	5, 20, 50 μg/mL	24 h or 48 h			
Gelan Xiaoke Pills	Pueraria lobata (Willd.) Ohwi, Trichosanthes kirilowii Maxim., Rehmannia glutinosa (Gaertn.) Libosch. ex Fisch. & C.A. Mey., Dioscorea opposita Thunb., Astragalus membranaceus (Fisch.) Bunge, Schisandra chinensis (Turcz.) Baill., Ophiopogon japonicus (L.f.) Ker Gawl., Anemarrhena asphodeloides Bunge	T2DM Complicated with Cardiovascular Diseases	Patients	one bag per time, three bags 1 day	Not mentioned	Alleviated insulin resistance	①④	(Wang)
Coptis root and ginseng formula	Coptis chinensis Franch., Panax ginseng C.A. Mey., Scutellaria baicalensis Georgi, Gardenia jasminoides J. Ellis, Phellodendron amurense Rupr., Glycyrrhiza uralensis Fisch. ex DC	T2DM	patients	11 g/d	3 months	Regulated blood sugar and gut microbiota	②	Jiang et al. (2022a)
Tang-ping-san	Astragalus membranaceus (Fisch.) Bunge, Anemarrhena asphodeloides Bunge, Pueraria lobata (Willd.) Ohwi, Trichosanthes kirilowii Maxim., Dioscorea opposita Thunb., Schisandra chinensis (Turcz.) Baill., Ophiopogon japonicus (L.f.) Ker Gawl., Rehmannia glutinosa (Gaertn.) Libosch. ex Fisch. & C.A. Mey.	T2DM	C57/BL6 mice	Not mentioned	28 days	Reduced the risk of hyperglycemia, insulin resistance, pathological organ changes, and inffammatory reactions	②	Yin et al. (2022)
Qijian mixture	Astragalus membranaceus (Fisch.) Bunge, Codonopsis pilosula (Franch.) Nannf., Atractylodes macrocephala Koidz., Poria cocos (Schw.) Wolf, Glycyrrhiza uralensis Fisch. ex DC., Angelica sinensis (Oliv.) Diels, Paeonia lactiflora Pall.	T2DM	KKay mice and C57/BL6 mice	1.795, 5.385 g/kg/d	8 weeks	Antidiabetic effects	②	Gao et al. (2018)
Jiang Tang San Huang pill	Coptis chinensis Franch., Scutellaria baicalensis Georgi, Astragalus membranaceus (Fisch.) Bunge, Pueraria lobata (Willd.) Ohwi, Trichosanthes kirilowii Maxim., Dioscorea opposita Thunb., Schisandra chinensis (Turcz.) Baill., Ophiopogon japonicus (L.f.) Ker Gawl., Rehmannia glutinosa (Gaertn.) Libosch. ex Fisch. & C.A. Mey.	T2DM	SD rats	0.27, 0.54, 1.08 g/kg	4 weeks	Ameliorated hyperglycaemia, insulin resistance, hyperlipidaemia, and pathological changes in the pancreas, liver, kidney and intestine and reduced the serum levels of pro-inflammatory cytokines	②	Tawulie et al. (2023)
Buyang Huanwu decoction	Astragalus membranaceus (Fisch.) Bunge, Angelica sinensis (Oliv.) Diels, Paeonia lactiflora Pall., Ligusticum chuanxiong Hort., Carthamus tinctorius L., Prunus persica (L.) Batsch, Pheretima aspergillum (E. Perrier)	Co-morbid T2DM and obesity	Zucker diabetic fatty (ZDF) rats and Zucker lean control (ZLC) rats	0.783 g/kg/d	7 weeks	Decreased the abnormally high blood glucose of high-fat diet-induced T2DM	②⑤	Liu et al. (2022a)
“maccog” traditional Chinese medicine (TCM) tea	Morus alba L., Astragalus membranaceus (Fisch.) Bunge, Zea mays L., Lycium chinense Mill., Gynostemma pentaphyllum (Thunb.) Makino, Ophiopogon japonicus (L.f.) Ker Gawl.	T2DM	patients	1,000– 1,500 mL every day, 6–7 days a week	12 weeks	Improved glucolipid metabolism and significantly increased the abundance of beneficial gut microbiota	②⑤	Hu et al. (2023)
Huoxue Jiangtang Decoction	Astragalus membranaceus (Fisch.) Bunge, Rehmannia glutinosa (Gaertn.) Libosch. ex Fisch. & C.A. Mey., Carthamus tinctorius L., Ophiopogon japonicus (L.f.) Ker Gawl., Rheum palmatum L., Prunus persica (L.) Batsch, Dioscorea opposita Thunb., Pseudostellaria heterophylla (Miq.) Pax ex Pax & Hoffm.	T2DM	SD rats	15.86 g/kg/day	8 weeks	Improved the metabolism of glycolipid and the function of β-cell	②⑤	Huang et al. (2022a)
Bupiwei Xieyinhuo Shengyang Decoction	Astragalus membranaceus (Fisch.) Bunge, Codonopsis pilosula (Franch.) Nannf., Bupleurum chinense DC., Actaea cimicifuga L., Atractylodes lancea (Thunb.) DC., Notopterygium incisum Ting ex H.T. Chang, Scutellaria baicalensis Georgi, Gypsum Fibrosum, Glycyrrhiza uralensis Fisch. ex DC.	T2DM	Hartley guinea pig	7.74 mL/kg/d	6 weeks	Reduced the risk of macroangiopathy	②	Li et al. (2024)
Bupleurum chinense DC. Guizhi Ganjiang decoction	*Lonicera japonica* Thunb., Scutellaria baicalensis Georgi, Ostreidae, Trichosanthes kirilowii Maxim., Cinnamomum cassia (L.) J. Presl, Zingiber officinale (Willd.) Roscoe, Glycyrrhiza uralensis Fisch. ex DC.	T2DM	Wistar rats	11.24 g/kg twice 1 day	8 weeks	Improved FBG and HbA1c	②	Yue and Jin (2022)
Fufang Zhenzhu Tiaozhi capsule	Coptis chinensis Franch., Salvia miltiorrhiza Bunge, Panax notoginseng (Burk.) F.H. Chen, Ligustrum lucidum W.T. Aiton, Cirsium japonicum Fisch. ex DC., Eucommia ulmoides Oliv., Citrus medica L. var. sarcodactylis (Hoola van Nooten) Swingle, Atractylodes macrocephala Koidz.	Diabetes-accelerated atheroscleros	ApoE^−/−^ mice	1.2, 2.4 g/kg	12 weeks	Improved glucolipid metabolic disorders, hypoglycemic and lipid-lowering effects and protection of vascular endothelial cell	③	Zhang et al. (2023)
Shengmai San	Panax ginseng C.A. Mey., Ophiopogon japonicus (L.f.) Ker Gawl., Schisandra chinensis (Turcz.) Baill.	Diabetic cardiomyopathy	SD rats	5 mL/kg	10 weeks	Reduced myocardial injury	④	Li (2022a)
Sanggua drink (SGD)	Morus alba L., Momordica charantia L., Pueraria lobata (Willd.) Ohwi, Dioscorea opposita Thunb.	T2DM	SD rats	1,240 mg/kg b.w	42 days	Increased hepatic glycogen Production and a hypoglycemic effect	⑤	Cai et al. (2018)
Danhong injection	Salvia miltiorrhiza Bunge, Carthamus tinctorius L.	Coronary heart disease angina pectoris and DM	patients	40 mL/d	6 months	Improved patient’s cardiac function and control angina attacks	④⑤	Li (2022b)
Tianhuang formula (THF)	Panax notoginseng (Burk.) F.H. Chen ex C.Y. Wu & K.M. Feng, Coptis chinensis Franch.	T2DM	Male C57BL/6 J Narl mice	60, 120 mg/kg/day	6 weeks	Regulated adipocyte mitochondrial function	⑤⑥	Luo et al. (2023)
Jinlida granules	*Panax ginseng* C.A. Mey., *Polygonatum odoratum* (Mill.) Druce, *Atractylodes lancea* (Thunb.) DC., *Sophora flavescens* Aiton, *Liriope* Herb., *Rehmannia glutinosa* (Gaertn.) Libosch. ex DC., *Polygonum multiflorum* Thunb., *Cornus officinalis* Siebold & Zucc., *Poria cocos* (Schw.) Wolf, *Eupatorium fortunei* Turcz., *Coptis chinensis* Franch., *Anemarrhena asphodeloides* Bunge, Epimedium Tourn. ex L., *Salvia miltiorrhiza* Bunge, *Pueraria thomsonii* Benth., *Litchi chinensis* Sonn., *Lycium chinense* Mill.	Diabetic cardiomyopathy	db/db mouse	1.75, 3.5 g/kg/d	8 weeks	Alleviated cardiac hypertrophy and myocardial inflammation, and decreased the expression of cardiac hypertrophy- and inflammation-related factors	⑥	Fang et al. (2024)
			AC16, H9C2 and HEK293 cells	50, 100, 200 μg/mL	Not mentioned			
Si wei jiang huang tang san	*Curcuma longa* L., *Phellodendron chinense* Schneid., *Tribulus terrestris* L., *Phyllanthus emblica* L.	T1DM	HepG2 cells	100, 200 μg/mL	24 h	Promoted glucose consumption	⑥	Xu et al. (2024)
			Male C57BL/6 N mice	61.25, 122.5, 225 mg/kg/d	7 days			
Simiao wan	*Phellodendron amurense* Rupr., *Atractylodes lancea* (Thunb.) DC., *Achyranthes bidentata* Blume, *Coix lacryma-jobi* L.	T2DM	Male C57BL/6 J	1.2 g/kg/d	8 weeks	Improved glucose tolerance, serum insulin, HDL-C, hepatocyte morphology, and liver glycogen synthesis	⑥	Xia et al. (2022)
Cortex mori-polygonatum odoratum tablets	*Morus* L. extract, *Polygonatum odoratum* (Mill.) Druce extract, *Hippophae rhamnoides* L. extract, *Curcuma longa* L. extract, Chromium rich yeast	T2DM	Patients	twice a day, take two tablets each time	2 months	Hypoglycemic effect	⑦	Chen (2022)
Banxiaxiexin decoction	*Pinellia ternata* (Thunb.) Makino, *Scutellaria baicalensis* Georgi, *Zingiber officinale* Roscoe, Panax ginseng C.A. Mey., *Glycyrrhiza uralensis* Fisch. ex DC., *Coptis chinensis* Franch., *Ziziphus jujuba* Mill.	T2DM cold-heat complicated syndrome	patients	100 mL each morning and evening	12 weeks	Hypoglycemic effect	⑦	Zhang et al. (2024b)
Liuwei Dihuang pills	*Rehmannia glutinosa* (Gaertn.) Libosch. ex DC.,*Cornus officinalis* Siebold & Zucc., *Dioscorea opposita* Thunb., *Poria cocos* (Schw.) Wolf.,*Alisma orientale* (Sam.) Juz., *Paeonia × suffruticosa* Andrews	T2DM qi-yin deficiency syndrome	patients	6 g/time, 2 times/d	1 month	Hypoglycemic effect	⑦	Zhu (2024)

①Reducing inflammation and regulating immunity. ②Regulation of Intestinal flora. ③Inhibiting apoptosis and enhancing cell function of islet cells or cardiovascular cells. ④Antioxidant Stress. ⑤Improving glucose and lipid metabolism. ⑥Other mechanisms. ⑦Not mentioned.

### 2.1 Reducing inflammation and regulating immunity

T1DM arises from an autoimmune attack on the pancreas, leading to the destruction of insulin-secreting β-cells. Immune system cells contribute to β-cell death through various mechanisms, including triggering inflammation. Some researchers describe T1DM as a “chronic anti-auto-inflammatory response,” and there is a theory suggesting that pancreatic inflammation may be the most important cause of T1DM (Committee, 2024; Gearty et al., 2022). Once T1DM develops, it can cause inflammation in other parts of the body, which may result from immune response to hyperglycemia (Qi et al., 2022). T2DM has a complex bidirectional relationship with inflammation. T2DM, characterized by IR, can lead to chronic inflammation, which further exacerbates IR, creating a vicious cycle. Chronic and systemic inflammation are the prominent features of T2DM. Excessive cytokines and signaling proteins generated to control inflammation can inadvertently lead to heightened inflammatory responses. In both T1DM and T2DM diabetes, chronic inflammation can lead to DACC such as cardiomyopathy, atherosclerosis, coronary heart disease, and other cardiovascular diseases (Lorenzo-Almoros et al., 2022; Peng et al., 2023; Poznyak et al., 2020). This underscores the rationale behind targeting inflammation or regulating the immune system as therapeutic strategies for DM. Several TCM compound prescriptions have demonstrated efficacy in addressing these issues. For example, GegenQinlian Decoction, BaihuRenshen Decoction, and Shen-Qi Compound Formula promote glucose absorption and exert hypoglycemic effects in diabetic rats by inhibiting small intestinal inflammation (Xu et al., 2020; Yao et al., 2022; Zhang and Liu, 2024). Jinlida granules and Danggui SiniSan can inhibit islet cell inflammation, enhance islet cell function, and play hypoglycemic roles (Gu et al., 2024; Wu, 2024). The Fufang Zhenzhu Tiaozhi capsule can reduce vascular endothelial inflammation and alleviate DACC (Wang L. et al., 2022). Gelan Xiaoke Pills can enhance the immune function in patients with diabetes, reduce IR, and alleviate diabetes symptoms (Wang M.-C. et al., 2024).

### 2.2 Regulation of intestinal flora

The intestinal flora is often referred to as the “hidden organ” of the human body, that is involved in regulating various biological processes such as energy metabolism and immune inflammatory response, and plays an irreplaceable role in the metabolic health of the body and the occurrence and development of diseases (Drozdz et al., 2021). Under normal physiological conditions, the gut maintains a complete immune barrier. However, this barrier is broken down once hyperglycemia occurs. An imbalance in the intestinal microecology is an important factor that accelerates the occurrence, development, and outcomes of various endocrine and metabolic diseases. Several studies have found that T2DM is often accompanied by intestinal flora disturbances and multiple organ dysfunctions (Mao et al., 2023; Nesci et al., 2023; Zhang and Xie, 2024). The intestinal leakage theory suggests that when the intestinal flora is disturbed, intestinal permeability increases, endotoxin and pro-inflammatory cytokine production increases, energy intake increases, and systemic inflammation and IR are induced (Cristofori et al., 2021; Gonzalez et al., 2019). The “gut-islet axis” is an important endocrine regulation axis of intestinal microecology and intestinal neuroendocrine dialogue with islets. Therefore, it is important to explore functional protective strategies for islet cells within the gut.

Several TCM compound prescriptions, including Gegen Qinlian decoction, Shen Qi compound formula, *Coptis* saliva root and *Ginseng* Alph. Wood formula, Tang-ping-san, Qijian mixture, JiangTang San Huang pill, Buyang Huanwu decoction, Chaihu Guizhi Ganjiang decoction, “maccog” TCM tea, Huoxue Jiangtang Decoction and Bupiwei Xieyinhuo Shengyang Decoction can increase the proportion of beneficial bacteria by regulating intestinal flora, and play a role in lowering blood glucose or alleviating diabetes symptoms (Gao et al., 2018; Hu et al., 2023; Huang Q. et al., 2022; Jiang L. et al., 2022; Liu M. et al., 2022; Tawulie et al., 2023; Wang B. et al., 2022; Zhang F. et al., 2024). From the perspective of TCM, the small intestine and the heart are similar to the “gut - spindle.” Long-term dietary changes in patients with pre-DM lead to a structural imbalance of the intestinal flora, which aggravates the changes in metabolic products (mainly trimethylamine oxide, short-chain fatty acids, bile acids, and lipopolysaccharides) of the flora (Bielka et al., 2022; Bondy, 2023). These substances can enter systemic circulation, damaging the intestinal barrier, and leading to complications such as myocardial fibrosis, vascular inflammation, and other diabetic cardiovascular diseases. This progression can result in poor management of the patient’s condition, uncontrollably and substantially accelerating mortality rates among patients with DM. Therefore, protecting the integrity of the intestinal barrier, improving imbalance of the intestinal flora, and regulating the metabolites of the flora can inhibit inflammatory responses in cardiomyocytes, delaying myocardial fibrosis, and protect cardiomyocytes. Such strategies represent novel auxiliary approaches for the treatment of DACC. Chaihu Guizhi Ganjiang Decoction has been shown to reduce blood glucose levels in diabetic rats by adjusting the absorption of short-chain fatty acids (Li et al., 2024). Bupiwei Xieyinhuo Shengyang Decoction exerts its effect by regulating the metabolites of intestinal flora to lower blood glucose (Yue et al., 2022).

### 2.3 Inhibiting apoptosis and enhancing the function of islet cells or cardiovascular cells

Islet cells form the basis of insulin secretion, and dysfunction in these cells, including the abnormal apoptosis of islet cells caused by various factors, occurs in most patients with DM. Therefore, the development of inhibitors of islet cell apoptosis is considered one of the most effective strategies for the prevention and treatment of DM. According to the TCM theory, jinlidagranules can tonify the spleen and push qi, a fundamental substance or driving force that sustains human life activities in the effect of TCM theory (Gu et al., 2024). Several studies have shown that jinlidagranules exert hypoglycemic effects by inhibiting islet cell apoptosis and enhancing islet function (Gu et al., 2024). Abnormal death of cardiovascular cells, such as cardiomyocytes and vascular endothelial cells, is a major cause of cardiovascular complications in patients with DM. Danggui SiniSan has the functions of nourishing blood and warming the meridian, namely, dispelling cold air in TCM theory (Wu, 2024). Standard biomedical treatment suggests that Danggui SiniSan can promote blood circulation by increasing vital energy, enhancing resistance, and regulating endocrine function. Some studies have shown that it not only enhances islet function by inhibiting islet cell apoptosis, but also protects cardiovascular function (Wu, 2024). Fufang Zhenzhu Tiaozhi capsules can inhibit endothelial cell apoptosis induced by DM, thereby safeguarding cardiovascular function in patients with diabetes (Wang L. et al., 2022; Zhang et al., 2023).

### 2.4 Antioxidant stress

Oxidative stress refers to an imbalance between oxidation and antioxidant activity, leaning toward oxidative damage. Oxidative stress is mainly involved in DM development through at least three ways: 1) blocking of the insulin action pathway, which leads to IR (Masenga et al., 2023), 2) reducing insulin gene expression, resulting in reduced insulin synthesis and secretion (Hoseini et al., 2022), and 3) promoting islet cell apoptosis (Zhao et al., 2023). Gel XiaokePills can enhance antioxidant capacity and thus reduce IR (Wang J. et al., 2024). In addition, hyperglycemia can induce or aggravate cardiovascular complications in several ways: 1) it directly increases reactive oxygen species (ROS) by aggravating mitochondrial load and the diabetic vascular inflammatory response (Ma X. M. et al., 2023), 2) non-enzyme-catalyzed glycosylation of proteins is enhanced during hyperglycemia. Therefore, the glycosylation of antioxidant enzymes, such as superoxide dismutase, inevitably leads to changes in enzyme activity, a decrease in free radical scavenging ability, induction of oxidative stress, and aggravation of DACC (Perrone et al., 2020), 3) hyperglycemia directly induces angiotensin production in muscle cells. Ang II can produce superoxide ions by activating the NADPH/NADH system (Zhang L. et al., 2024). Induction of oxidative stress results in vascular endothelial cell dysfunction and cardiovascular diseases, and 4) it promotes the proliferation and migration of vascular smooth muscle cells (Chen J. W. et al., 2020). Previous studies have shown that the proliferation and migration of these muscle cells play an important role in the pathogenesis of atherosclerosis (Gong X. et al., 2021; Yan Y. et al., 2021). Recent studies have used TCM to treat different cell types and alleviate DM by affecting the mitochondrial oxidative respiratory chain, showing that oxidative stress has a bidirectional regulatory effect on DACC (Gong X. et al., 2021; Zhang Y. et al., 2024). ShengmaiSan is a combination of three important components that alleviate DM-induced myocardial damage by improving oxidative stress response (Li Y.-Y., 2022). Danhong injection has been reported to improve myocardial function in patients with DM through its anti-oxidative stress properties (Li D. L., 2022).

### 2.5 Improving glucose and lipid metabolism

Abnormal glucose metabolism is a well-known underlying cause of DM. The metabolic abnormalities that lead to DM include reduced glycogen synthesis and increased glycogen breakdown. Sangguadrink directly lower blood sugar levels by promoting liver glycogen synthesis (Cai et al., 2018). There exists a close relationship between glucose and lipid metabolism. When adipose tissue absorbs glucose, fat synthesis diminishes. Simultaneously, the mobilization and decomposition of stored fat are accelerated, resulting in a heightened level of free fatty acids and triglycerides in the bloodstream. Elevated levels of fatty acids can inhibit the synthesis of liver glycogen, promoting the production of more glucose and further aggravating hyperglycemic symptoms. Moreover, high concentrations of free fatty acids and triglycerides in the bloodstream increase the susceptibility of the myocardium to ischemic damage, thus inducing or aggravating DACC (Park, 2021). Lipid metabolism disorders result in fat accumulation in the liver, muscles, and blood vessel walls, affecting the normal role of insulin and resulting in IR, which is more likely to induce or aggravate hyperglycemia (Yan B. F. et al., 2023). Therefore, a research team found that Buyang Huanwu decoction can reduce the symptoms of T2DM in mice on a high-fat diet by improving lipid metabolism (Liu M. et al., 2022). Similar to the results of “maccog,” a kind of TCM tea, can improve the glucolipid metabolism and improve the symptoms of T2DM (Hu et al., 2023). In addition, HuoxueJiangtang decoction could enhance the function of islet β-cells by improving glucose and lipid metabolism (Huang Q. et al., 2022). Danhong injection, a certified Chinese medical product made from *Salvia miltiorrhiza* Bunge and *Carthamus tinctorius* L., is prescribed to patients with coronary heart disease in China and can improve cardiac function and control angina attacks by improving glucolipid metabolism (Li D. L., 2022). The Tianhuang formula can improve the mitochondrial function of adipocytes by regulating glycolipid metabolism and alleviating diabetes symptoms (Luo et al., 2023).

### 2.6 Other mechanisms

The development and progression of DACC involve multiple genes and signaling pathways (Graczyk et al., 2024; Wang H. et al., 2024). Jinlid granules may mitigate DACC by modulating signal transduction or the TP53 pathway (Fang et al., 2024; Gu et al., 2024). Tianhuang formula improves glycolipid metabolism in diabetic mice through the AMPK/MICU1 pathway (Luo et al., 2023). Si wei jiang huang tang san promotes glucose consumption by activating the ERK signaling pathway and inhibiting HIF-1α, so as to alleviate the symptoms of T1DM (Xu et al., 2024). Furthermore, Simiao Wan improved glucose tolerance, serum insulin, high density lipid cholesterol, hepatocyte morphology, and liver glycogen synthesis in T2DM mice by regulating the insulin receptor substrate-1/AKT2/FOXO1/glucose transported type (GLUT) 2 pathway (Xia et al., 2022).

Endothelial-mesenchymal transition (EndMT) refers to the process by which endothelial cells lose their original characteristics and transform into mesenchymal cells (myofibroblasts and smooth muscle cells) under the action of various stimulus factors. This transformation results in significant changes in their polarity, morphology, and function of endothelial cells. In the context of diabetes, vascular endothelial cells promote fibrosis after EndMT treatment, subsequently becoming more permeable to promote white blood cell and lipid accumulation in the arterial intima, resulting in plaque formation (You et al., 2022). Fufang Zhenzhu Tiaozhi capsules have been shown to inhibit EndMT and prevent or reduce cardiovascular complications associated with diabetes (Wang L. et al., 2022).

In addition, several other TCM prescriptions, such as Cortex mori-polygonatum odoratum tablets, Liuwei Dihuang Pills (Tang), and Banxiaxiexin Decoction, are used to prevent or treat DACC; however, the specific mechanism of this treatment remains unclear (Chen, 2022; Zhang F. et al., 2024; Zhu, 2024). Therefore, elucidating the specific mechanisms of action is essential for future studies.

## 3 Application of TCM botanical drugs in DACC

TCM botanical drugs are composed of plant extracts and are widely used in treatment, healthcare, skincare, and other fields because of their natural and nontoxic characteristics. In recent years, an increasing number of studies have shown that many botanical TCM drugs and their extracts play crucial roles in combatting DACC (Prasopthum et al., 2022; Zhang L. et al., 2024; Zhou G. et al., 2023). TCM botanical drugs extracts from different solvents can prevent and treat DACC via multiple mechanisms (Table 4). These mechanisms mainly include reducing inflammation and regulating the immune system; properly regulating the intestinal flora; inhibiting the apoptosis or death of islet and cardiovascular cells, thereby enhancing their functions; affecting cellular stress, including endoplasmic reticulum stress (ER) and oxidative stress; and regulating glucose and lipid metabolism, thereby reducing IR.

**TABLE 4:** Lists of TCM botanical drugs with potential anti-diabetes and its cardiovascular complications action.

TCM botanical drugs	Extraction solution	Diseases	Model	Optimal dose	Duration of treatment	Effects	Potential mechanism	References
Propolis	Ethanol	T2DM	C57BL/6 mice	600 mg/kg·bw	4 weeks	Reduced fasting blood glucose	①②⑤	Guan et al. (2023)
*Citrus reticulata* Blanco and *Lycium China* Mill.	Ethanol	T2DM	SD rats	400 mg/kg/d	4 weeks	Normalized blood pressure and the plasma lipid profile as well as the plasma levels of liver enzymes	①	Wang et al. (2022c)
*Lycium chinense* Mill.	Ethanol	Diabetic cardiomyopathy	SD rats	100, 400 mg/kg	5 weeks	Cardioprotective effects	①③④	Wen et al. (2022a)
*Pueraria montana* (Lour.) Merr. and Hippophae rhamnoides L.	Aqueous	T2DM	db/db mice	1.34 g/kg, 0.89 g/kg, 0.45 g/kg/d	8 weeks	Alleviated the symptoms of T2DM mellitus	①⑤	Liu et al. (2024)
*Sanghuangporus vaninii* (Ljub.) L.W. Zhou & Y.C. Dai	Ethanol	T2DM	ICR mice	100, 300 mg/kg/d	4 weeks	Improved body weight, glycolipid metabolism, and inflammation-related parameters	②	Huang et al. (2022a)
*Zingiber officinale* Roscoe	Ethanol	T2DM	Kunming mice	25 mg/kg 0.2 mL, 50 mg/k 0.2 mL, 100 mg/kg 0.2 mL	4 weeks	Prevented severe insulin resistance in mice	②	Chen (2022)
*Anemarrhena asphodeloides* Bunge extract (AAE)	Ethanol	T2DM	Wistar rats	20, 60, 180 mg/kg/d	4 weeks	Restored pancreatic islet cell function	②	Yan et al. (2021a)
*Phellinus baumii* Pilát	Ethanol (EE)	T2DM	HepG2 cell	EE: 50, 100 μg/mL AE: 100 μg/mL	24 h	Hypoglycemic activity	②⑤	Zheng et al. (2023)
	Aqueous (AE)		ICR mice	EE: 50, 100 mg/kg AE: 50 mg/kg				
*Dendrobium officinale* Kimura & Migo extract	Not mentioned	T2DM	BKS.Cg-Dock7m +/+Leprdb/Nju mice	1 g/kg/day	30 days	Reduced the fasting blood glucose levels	②	Wang et al. (2021)
*Pueraria montana* (Lour.) Merr.	Ultra-pure water	T2DM	db/db mice	0.89 g/kg/d	8 weeks	Ameliorated the T2DM symptoms, metabolic disorder	②	Li et al. (2023a)
*Cassia tora* L. seeds	Ethanol	T2DM	βTc3 cell	15, 30 mg/L	24 h	Reduced the inhibition rate and apoptosis rate of β Tc3 cells	③	Bai et al. (2020)
*Morus alba* L. leaves	Deionized water	T2DM	Wistar rats	4.0 g crude drug/kg p.o. Daily	8 weeks	Increased insulin sensitivity and improved glycemic control	③	Shi et al. (2023)
*Cynara scolymus* L.	Hot water	Insulin resistant T2DM	IR in HepG2 cells	Not mentioned	24 h	Improved glucose metabolism	④⑤	Deng et al. (2023)
*Trichosanthes* L.	Pure water	T1DM	SD rats	200 mg/kg/day	5 weeks	Regulated the diabetes-induced lipid metabolism disorder, increaed the levels of insulin and C-peptide, and alleviated the symptoms of diabetes	④	Zhang et al. (2022)
	Anhydrous ethanol			20 mg/kg	5 weeks		⑤	
*Astragalus membranaceus* Fisch. ex Bunge	Aqueous	High glucose induced endothelial cell damage	HUVEC	Mangiferin (26, 13, 6.5 μmol/L), daidzein (7.00, 3.5, 1.75 μmol/L)	24 h	Protected HUVEC damage induced by high glucose	④	Ma et al. (2024)
*Garcinia cambogia* Desr.	Distilled water	HFD-induced IR	C57BL/6 mice	0.2, 1, 4, 5 g/L	16 weeks	Inhibited the HFD-induced hepatic lipid accumulation	⑤	Dong et al. (2023)
*Siraitia grosvenorii* (Swingle) C. Jeffrey ex A. M. Lu and Zhi Y. Zhang	Ethanol + distilled water	T2DM	SD rats	0.505, 0.343, 0.056, 0.040, 0.051, 0.020 g/kg.d.bw		Anti-hyperglycemic	⑤	Zhang et al. (2020)
*Hippophae rhamnoides* L.	Ethanol	Hyperglycemic	Kunming mice	1.5 g/kg	5 weeks	Hypoglycemic action	⑤	Yan et al. (2023b)
			Caco-2 cells	6.25–100 μg/mL	24 h			
Glucidum lucidum (Leyss. ex Fr.) Karst.	Ethanol	Insulin resistance T2DM	3T3-L1 Adipocytes	10, 50, 100 mg/L	24 h	Increased glucose consumption and intracellular triglyceride content of adipocytes	⑤	Tan et al. (2022)

①Reduce inflammation and regulate the immune system. ②Regulate the Intestinal Flora Properly. ③Inhibit the Apoptosis or Death of Islet Cells and Cardiovascular Cells, and Enhance Their Functions. ④Affect Cellular Stress, including Endoplasmic Reticulum Stress and Oxidative Stress. ⑤Regulate glucose and lipid metabolism, and reduce insulin resistance.

### 3.1 Reducing inflammation and regulating immunity

Studies have found that propolis alcohol extract and Propolis water extracts can alleviate IR by reducing inflammation and regulating glucose metabolism, thereby alleviating DM symptoms (Guan et al., 2023). Inflammation also plays an important role in DACC (Spinetti et al., 2023). *Citrus reticulata* Blanco and *Lycium Chinense* Mill. alcohol extracts can inhibit vascular endothelial and myocardial cell inflammation, thus preventing or easing DACC (Wang Y. et al., 2022; Wen et al., 2022a).

### 3.2 Regulation of gut flora

Gut flora also plays an important role in DACC (Alka et al., 2022). *Pueraria montana* (Lour.) Merr. aqueous extract and *Sanghuangporus vaninii* (Ljub.) L.W. Zhou and Y.C. Dai, *Zingiber officinale* Roscoe, *Anemarrhena asphodeloides* Bunge (AAE) alcohol extract, *Phellinus baumii* Pilát water and alcohol extract, and *Dendrobium officinale* Kimura and Migo extract, *Pueraria montana* (Lour.) Merr. water extracts can improve IR and blood sugar levels by regulating intestinal flora (Chen, 2022; Huang Z. R. et al., 2022; Li J. et al., 2023; Wang et al., 2021; Yan D. et al., 2021; Zheng et al., 2023; Zhu et al., 2024).

### 3.3 Inhibiting islet or cardiovascular cell death and enhancing cell function

Cell death usually occurs in various forms, such as apoptosis, necroptosis, pyroptosis, ferroptosis, and other forms discovered so far. The death of islets and cardiovascular cells is one of the major contributing factors to DACC. In recent years, TCM botanical drugs have gained popularity as treatment for DACC. The alcohol extract of *Lycium chinense* Mill. was used to treat diabetic cardiomyopathy in rats (Wen et al., 2022a). The results showed that inhibiting the apoptosis of heart cells is one of the main mechanisms for improving diabetic cardiomyopathy. *Cassia obtusifolia* L. is commonly used in clinical medicine to treat eye diseases, constipation, hypertension, hyperlipidemia, and DM. Studies have found that the use of *Cassia tora* L. seeds in the treatment of DM is mainly related to the inhibition of islet cell apoptosis (Bai et al., 2020). Ferroptosis, a new type of programmed cell death that is iron-dependent and differs from apoptosis, cell necrosis, and autophagy, was first proposed by Dr. Brent R. Stockwell of Columbia University in 2012 (Zeng et al., 2023). *Morus alba* L. leaves, an herb with high medicinal and economic value, possesses an aqueous extract capable of inhibiting ferroptosis in islet cells (Shi et al., 2023).

### 3.4 Effects of cellular stress (ER and oxidative stress)

The ER plays an important role in protein folding and is highly sensitive to changes in cellular homeostasis. Changes in the environment in which proteins fold can lead to the aggregation of unfolded or misfolded proteins and affect normal cell function (Chen and Zhang, 2023). When the ER is stressed, the unfolded protein response alleviates protein misfolding and restores cell homeostasis through a series of adaptive responses or induces apoptosis if homeostasis cannot be reshaped (Wiseman et al., 2022). ROS production has been confirmed to be closely related to ER stress and the unfolded protein response (Zeeshan et al., 2016). Although ROS are toxic, they can also mediate physiological processes as messenger molecules. The cytoplasm and various organelles, including the ER and mitochondria, produce ROS (Liu X. et al., 2022). Changes in the redox state of the ER can cause ER stress, which in turn induces ROS in the ER and mitochondria. Sustained oxidative and ER stress initiate apoptosis (Sahu et al., 2023). *Lycium chinense* Mill. attenuated cardiac oxidative stress and protected the myocardium (Wen et al., 2022a). *Cynara scolymus* L. improves IR in HepG2 cells by inhibiting ER stress (Deng et al., 2023). *Astragalus membranaceus* Fisch. ex Bunge and *Trichosanthes* L. aqueous extracts improve DACC through their anti-oxidant effects (Ma et al., 2024; Zhang et al., 2022).

### 3.5 Regulation of glucose and lipid metabolism and reduction in IR

Several botanical TCM drugs do not depend on a single mechanism of action to exert their effects. For example, Propolis ethanol extracts and *Phellinus baumii* Pilát have been shown to reduce IR by regulating glucose and lipid metabolism (Spinetti et al., 2023; Zheng et al., 2023). Different extraction solvents may exhibit different mechanisms of action. In addition to the antioxidant activity of the alcohol extract, *Trichosanthes* L. water extract improved the symptoms of type 1 diabetic rats by improving the liver glycogen content (Zhang et al., 2022). *Garcinia cambogia* Desr. aqueous extracts alleviate diabetes symptoms by improving lipid metabolism (Dong et al., 2023). Water extract of *Siraitia grosvenorii* (Swingle) C. Jeffrey ex A. M. Lu andand Zhi Y. Zhang promotes Glucagon-like peptide-1 (GLP-1) secretion, reduces insulin secretion, and has a hypoglycemic function. *Hippophae rhamnoides* L. is a plant that shares medicinal and dietary roles, has an alcohol extract that inhibits glucose absorption in the small intestine (Zhang et al., 2020). *Glucidum lucidum* (Leyss. ex Fr.) Karst. a type of fungus that reduces IR by increasing glucose consumption and intracellular triglyceride content in fat cells (Tan et al., 2022). Hypoglycemic effects of *Pueraria montana* (Lour.) Merr. and *Hippophae rhamnoides* L. are associated with reduced IR (Liu et al., 2024; Yan C.-Y. et al., 2023).

## 4 Application of TCM monomers in DACC

Natural pharmaceutical chemicals consist of both inorganic and organic components. In TCM, the content of inorganic components such as minerals and metals, is relatively low compared with organic components Alkaloids, saponins, polysaccharides, flavonoids, phenanthrene quinones, phenols, and terpenoids are important organic components. Notably, compounds belonging to the same class can exhibit different mechanisms of action. The mechanisms of action of these organic compounds in DACC include: 1) reducing inflammation and regulating immunity; 2) regulating intestinal mass; 3) inhibiting apoptosis or death of islet cells or cardiovascular cells; 4) affecting cellular stress (ER and oxidative stress); 5) regulating glycolipid metabolism and alleviating IR; 6) other mechanisms (affecting cell microenvironment, ion channels, and activation of autophagy) (Table 5).

**TABLE 5:** Lists of TCM monomers with potential anti-diabetes and its cardiovascular complications action.

Types of compounds	Compounds	Diseases	Model	Optimal dose	Duration of treatment	Effects	Potential mechanism	References
Alkaloid	Berberine	T2DM	GK rats and Wistar rats	200 mg/kg	12 weeks	Attenuated intestinal inflammation and lowered glucose	①②	Xu et al. (2020)
		T2DM	Patients	300 mg	12 weeks	Anti-diabetic	①	Nematollahi et al. (2022)
	Matrine	T2DM	Mouse intestinal secretory cell line (STC-1)	0, 0.2, 0.5, 1, 5, 10, 15, 20 mmol/L	24, 48, 72 h	Lowered blood sugar	⑤	Gao et al. (2018)
		Diabetic cardiomyopathy	SD rats	200 mg/kg/d	10 days	Attenuated cardiac fibrosis	④	Liu et al. (2017)
		Diabetic cardiomyopathy	SD rats	5 mg/kg, once a day	10 weeks	Decreased nonfasting blood glucose levels and improved hemodynamic parameters	④	Hou et al. (2013)
Saponins	Astragaloside IV(AS-IV)	T2DM	Kunming mice	25, 50, 100 mg/kg, once daily	10 weeks	Reversed the abnormalities in blood lipids, glucose, insulin resistance, as well as oxidative stress levels	②④⑤	Gong et al. (2021a)
		T2DM	The mouse insulinoma Min6 cells	0, 12.5, 25, 50 μmol/L	26 h	Protected uric acid-induced pancreatic β-Cell dysfunction	③⑥	Li et al. (2024)
		Diabetic cardiomyopathy	Wistar rats	40 mg/kg/d	12 weeks	Improve the cardiac function	⑤	Wu et al. (2023a)
		Diabetic cardiomyopathy	C57BL/6 J mice	3, 6, 12 mg/kg	8 weeks	Inhibited endothelial dysfunction	④	Zhang et al. (2021)
	The fruits of *Sophora flavescens* Aiton (GFS)	Diabetic cardiomyopathy	Wistar rats	40, 80 mg/kg	12 weeks	Enhanced the secretion index of pancreatic beta cells and improved lipid metabolism disorders	①④	Sun (2020)
	Anemoside B4	T2DM	SD rats	2.5, 5 mL/kg	2 weeks	Improved hyperglycemia	⑤	Gong et al. (2023)
		T2DM	Male SD rats	5 mL/kg, twice 1 day	2 weeks	Improved hyperglycemia	⑤⑥	Gong (2023a)
Polysaccharides	*Dendrobium officinale* Kimura and Migo polysaccharide	T2DM	Male C57BL/6 mice	100, 200, 400 mg/kg	4 weeks	Relieved symptoms of high blood sugar	⑤	Liu (2019)
	*Lycium barbarum* Mill. polysaccharide	T2DM	C57BL/6 mice	50, 100, 200 mg/kg	6 weeks	Reduced blood glucose levels and improved insulin sensitivity	②	Ma et al. (2022)
	*Polygonatum sibiricum* Redouté polysaccharide (PP)	T2DM	db/db mice	1.0 g/kg	6 weeks	Hypoglycemic effect	②④⑤	Sun et al. (2019)
	Apurified RG polysaccharide (RGP) extracted from *Rehmannia* Libosch. ex Fisch. and C.A.Mey	T1DM	Kunming mice	20, 40 and 80 mg/kg	4 weeks	Anti-diabetic properties	⑤	Zhou et al. (2015)
	*Coix lacryma* L. polysaccharides	T2DM	C57BL/6J mice	175, 350 mg/kg	4 weeks	Hypoglycemic effect	②	Xia et al. (2021)
	*Polygonatum sibiricum* Redouté polysaccharide (APS)	Diabetic cardiomyopathy	SD rats	1 g/kg/d	16 weeks	Inhibited cardiomyocyte apoptosis	④	Chen et al. (2023)
(Iso)Flavonoids	Mangiferin	Diabetic cardiomyopathy	SD rats	20 mg/kg	16 weeks	Mitigated diabetic cardiomyopathy	⑥	Hou et al. (2013)
		Diabetic cardiomyopathy	SD rats	20 mg/kg	16 weeks	Ameliorated hyperglycemia, lowered left ventricular systolic pressure, and reduced apoptosis rate	①③④	Jin and Arroo (2023)
	Puerarin	Diabetes-associated cardiovascular disease	SD rats	100, 150 mg/kg	21 days	Exerted a protective effect on HUVECs and diabetic vasculopathy	③④	Wei et al. (2024)
		Myocardial injury in diabetes	SD rats	20, 40 mg/kg	5 weeks	Improved insulin resistance and myocardial injury	①③④	Kong et al. (2023)
	Luteolin	Diabetic cardiomyopathy	SD rats	5, 10 and 20 mg/kg	5 weeks	Alleviated cardiac pathological changes such as cardiac remodeling, inflammation and oxidative stress, improved cardiac function	①④	Chen (2023)
	The flavonoid component (FC) of *Agrimonia pilosa* Ledeb. (Rosaceae)	Adipocytes IR	C57BL/6 mice	1.0, 2.5, 5.0, 7.5, 10.0 mg/kg/d	4 weeks	Improved glucose metabolism	①④	Guo et al. (2023)
	Naringenin	T1DM with myocardial injury	C57BL/6	25, 50, 75 mg/kg	6 weeks	Reduced blood sugar and improved myocardial injury	④	Li et al. (2020b)
	Total flavonoids of *Murraya paniculata* (L.) Jack leaves (TFMP)	Diabetic cardiomyopathy	Wistar rats	40, 80 mg/kg	12 weeks	Enhanced the secretion index of pancreatic beta cells, improved lipid metabolism disorders	①④	Sun et al. (2020)
Phenanthraquinone	Tanshinone IIA	Diabetic cardiomyopathy	SD rats	2, 4 mg/kg	6 weeks	Improved cardiac pathological changes	③④	Tao et al. (2019)
			C57BL/6J mice	10, 50 mg/kg/day	2 months	Alleviated the pathological changes in the hearts of diabetic mice, ameliorated the cytopathological morphology of cardiomyocytes, reduced the cell death rate	③④	Wu et al. (2023a)
	Tanshinone I	T2DM	SD rats	30, 60, 120 mg/kg	3 weeks	Reduced blood glucose levels, and an alleviated insulin resistance	⑤	Wu et al. (2023b)
Phenolic compounds	Curcumin	T2DM	Kunming mice	50 mg/kg	4 weeks	Prevented the rise of blood sugar, reduced insulin resistance and improved insulin sensitivity, lowered the contents of TC and LDL-C, and increased the content of HDL-C; increased the activity of GSH-Px and reduced the contents of MDA, TNF-α and CRE	①④⑤⑥	Chen et al. (2022)
		Diabetic Cardiomyopathies	New Zealand rabbits	300 mg/kg/d	3 months	Increased nuclear transfer of Nrf2 and the expression of Gpx4 and HO-1, reduced glucose induced myocardial cell damage, and reversed myocardial cell damage caused by the ferroptosis inducer erastin	③④⑥	Wei et al. (2022a)
			Rat H9C2 cardiomyocytes	Not mentioned	24 h			
	RPE, a polyphenol-enriched extract of *Rosa rugosa* Thunb. (Rosaceae)	T2DM	SD rats	37.5, 75, 150 mg/kg bw	4 weeks	Improved glycogen synthesis and blood glucose regulation	⑤	Liu et al. (2017)
	Resveratrol	Heart microvascular injury in diabetes	Primary rat CMECs	2 μmol/L	48 h	Attenuated diabetic cardiac microvascular injury	④	Cai (2024)
Terpenoids	Diterpenoid components	IR hepatocytes	HepG2	50, 200 μMol/L	24 h	Hypoglycemic activity	③	Sun (2020)
	Euscaphic acid, Tormentic acid, Corosolic acid, Maslinic acid, Oleanolic acid, Ursolic acid	Not mentioned	Alpha glucosidase	Not mentioned	Not mentioned	Inhibited alpha glucosidase activity	⑤	Chen et al. (2020b)
Others	TPX,derived from the mangiferin derivative 1,3,6,7-tetrapropylene acyloxy ketone	IR hepatocytes	HepG2 and normal hepatocytes HL7702 cell	8.75, 17.5, 35, 70, 140, 280 μM	24 h	Restored the insulin signaling pathway, increased liver glycogen synthesis, and potentially protects insulin-resistant hepatocytes from glucose metabolism disorders	⑤	Fan et al. (2023)

①Reduce inflammation and regulate the immune system. ②Regulate the intestinal flora. ③Inhibit the apoptosis or death of islet cells or cardiovascular cells, and enhance cell function. ④Affect cellular stress (endoplasmic reticulum stress and oxidative stress). ⑤Regulate glucose and lipid metabolism, and reduce insulin resistance. ⑥Other mechanisms (affecting the cellular microenvironment, ion channels, activating autophagy, and affecting signal pathways).

Berberine is an isoquinoline alkaloid found in plants such as *Coptis* Salisb. and *Phellodendron* Rupr. Clinically, its hydrochloride form, known as berberine hydrochloride, is widely used to treat intestinal infections. With the deepening of pharmacological research, berberine hydrochloride has been reported to have numerous pharmacological effects, including anticancer, antitumor, bactericidal, antibacterial, anti-inflammatory, antioxidant, hypoglycemic, and lipid-lowering properties (Song et al., 2020). Early studies have demonstrated that berberine not only shows potential in hypoglycemic studies in animals and humans but also has considerable therapeutic effects in DACC (Coppinger et al., 2024). In addition, studies have shown that berberine can improve diabetic complications without causing substantial side effects (Chang et al., 2015; Dai et al., 2021). Compared with other first-line drugs and treatments, berberine is relatively inexpensive and suitable for long-term management of T2DM and related complications. Numerous studies have shown that berberine can considerably reduce blood sugar in rats by regulating the intestinal flora, alleviating intestinal inflammation, and increasing the absorption and utilization of glucose in the small intestine (Nematollahi et al., 2022; Xu et al., 2020). Furthermore, berberine can improve cardiometabolic status and myocardial inflammation, as well as improve blood sugar levels and protect the myocardium of diabetic patients (Nematollahi et al., 2022; Xu et al., 2020).

Matrine, an alkaloid extracted from the dried roots, plants, and fruits of *Sophora flavescens* Aiton, has a wide range of physiological activities, including antibacterial, antiviral, antioxidant, anti-inflammatory, immunomodulatory, anti-tumor, anti-fibrosis, and protective effects on multiple organs and tissues (Lin et al., 2022). As one of the effective active compounds, matrine increases insulin sensitivity, lowers blood sugar levels, and ameliorates DACC. IR can lead to impaired endothelial function, including barrier dysfunction, impaired nitric oxide (NO) activity, excessive production of ROS, oxidative stress, and inflammatory dysregulation. NO acts through eNOS-regulated biosynthesis, and is a potent vasodilator and an important vascular endothelial protective factor. The PI3K/Akt/eNOS signaling pathway is widely present in vascular endothelial cells and plays an important role in the regulation of vasodilation and contraction (Wen et al., 2022b). Activation of the PI3K/Akt signaling pathway enhances eNOS expression and plays a regulatory role in eNOS. Astragaloside IV activates the PI3K/Akt/eNOS signaling pathway and promotes eNOS expression to improve myocardial function in diabetic rats (Wu S. et al., 2023). Astragaloside IV can also enhance pancreatic beta cell dysfunction induced by uric acid through the activation of the PI3K/AKT pathway, anti-apoptotic effects, and activation of autophagy (Jiang Z. et al., 2022). Astragaloside IV can regulate intestinal flora, remove ROS free radicals, and reverse abnormal levels of blood lipids, blood sugar, IR, and antioxidant stress in Kunming mice (Gong P. et al., 2021; Zhang et al., 2021). Anemoside B4, a natural saponin extracted from Pulsatilla chinensis of the goldenseal family, has good efficacy as anti-tumor, anti-inflammation, and neuroprotective effects (Li Y. F. J. et al., 2020). Recent studies have found that Anemoside B4 also plays a role in lowering blood glucose levels by promoting glucose uptake by muscle cells, facilitating the transport and utilization of grape sleeves, enhancing GLUT4 expression, and regulating various mechanisms of the PI3K/AKT pathway (Gong, 2023a; Gong, 2023b; Gong et al., 2023). These studies provided data for the prevention and treatment of DM and its associated cardiovascular complications.

Polysaccharides from botanical drugs in TCM are complex sugars with complex molecular structures that are extracted from botanical drugs. An increasing number of studies have shown that polysaccharides have considerable efficacy in treating cardiovascular complications in diabetes (Zhang et al., 2019). *Dendrobium offiHerbalcinale* Kimura and Migo polysaccharides may regulate glycogen synthesis and glucose metabolism through the insulin/PI3K/Akt signaling pathways, improve sugar metabolic disorder in mice with T2DM, and exert hypoglycemic activity (Liu, 2019). The *Lycium Chinense* Mill. polysaccharide was derived from the medicine-edible plant *Lycium Chinense* Mill. LBP also reduces blood sugar levels and improves insulin sensitivity in diabetic mice by regulating gut flora (Ma et al., 2022). The purified RG polysaccharide was extracted from *Rehmannia* Libosch. ex Fisch. and C.A.Mey. can also lower blood sugar levels by affecting glucose metabolism (Zhou et al., 2015). Coix lacryma L., a TCM, has many health benefits, and *Coix lacryma* L. polysaccharides are its main active compounds. In a mouse model of DM, *Coix lacryma* L. polysaccharides were found to reduce blood sugar levels by improving the intestinal flora (Xia et al., 2021). Polysaccharides from *Polygonatum sibiricum* Redouté have a substantial hypoglycemic effect in T2DM (Chen et al., 2023). Plant polysaccharides not only have significant efficacy in DM but also show good prospects for the prevention and treatment of cardiovascular diseases. *Astragalus* L. inhibited cardiomyocyte apoptosis in diabetic mice by improving ER stress, providing strong evidence for the prevention and treatment of diabetic cardiomyopathy (Sun et al., 2019).

Flavonoids are a class of natural compounds characterized by a 2-phenylchromogen (flavone) structure. They can be classified into various structural types depending on the degree of oxidation of the central carbon, ring formation, and the junction site of the B ring. These structural types include flavones, isoflavones, flavonols, dihydroflavones, dihydroflavonols, dihydroisoflavones, chalcones, aurones, flavanes, anthocyanidins, anthocyanidins, and biflavones. Flavonoids are widely distributed in nature, and exhibit various biological activities. Many studies have shown that they have high application value in DM and cardiovascular diseases (Hou et al., 2013; Jin and Arroo, 2023).

Mangiferin, a compound derived from mangoes, possesses various pharmacological and nutritional properties. Upregulation of MMP-2 and downregulation of MMP-9 reduce myocardial collagen accumulation, protein expression of IRE1, ASK1, and JNK in cardiomyocytes, and ER stress in cardiomyocytes, thus inhibiting the progression of diabetic cardiomyopathy (Hou et al., 2013; Jin and Arroo, 2023). Puerarin, a derivative extracted from TCM, plays an active anti-inflammatory, anti-oxidation, anti-myocardial hypertrophy, and anti-myocardial fibrosis and is closely related to programmed cell death. Studies have shown that puerarin can improve or prevent cardiomyopathy in diabetic rats by inhibiting ROS production, pyroptosis, and the inflammation of cardiomyocytes and vascular endothelial cells (Bai et al., 2021; Wei et al., 2024). Luteolins are a class of flavonoids widely found in TCM botanical drugs and are natural antioxidants. Chen et al. found that luteolin reduced oxidative stress and myocardial tissue inflammation, thereby improving myocardial function in rats with diabetic cardiomyopathy (Chen, 2023). Flavonoid components of *Agrimonia pilosa* Ledeb (Rosaceae) alleviates oxidative stress injury through the c-Jun amino-terminal kinase (JNK)/PI3K/Akt pathway, reduces the expression and secretion of inflammatory cytokines while improving glucose metabolism, and alleviating fatty IR (Guo et al., 2023). Naringin is derived from the Rutaceae plant, *Citrus maxima* (Burm.) Merr. fruit and is a natural flavonoid compound. Grapefruit is a popular fruit worldwide (Li Y. F. J. et al., 2020). However, it is also a TCM that can serve as both medicine and food in China and has a variety of biological activities, including promoting digestion, anti-inflammatory effects, and relieving hangovers. The total flavonoids in *Murraya paniculata* (L.) Jack leaves relieve inflammation mediated by free-radical lipid peroxidation, strengthen the pancreatic beta cell secretion index, improve lipid metabolism disorders, and diabetic cardiomyopathy (Sun, 2020).

Phenanthraquinone is a quinone containing compound. Several studies have indicated that tanshinone IIA is the most fat-soluble component of *Salvia miltiorrhiza* Bunge, which can improve the symptoms of diabetic cardiomyopathy by inhibiting cardiomyocyte apoptosis mediated by ER stress (Tao et al., 2019; Wu S. et al., 2023). Tanshinone I is a natural phenanthrene quinone extracted from Salviorrhiza that can regulate glycogen metabolism and improve blood sugar levels and IR in T2DM rats (Wei et al., 2017).

Phenolic compounds are a diverse group of natural compounds commonly found in TCM. Curcumin is a natural polyphenolic compound extracted from ginger plants, which imparts a unique color and flavor to food. In addition, curcumin has significant medicinal value as it can improve human immunity, accelerate body metabolism, and have anti-inflammatory, antibacterial, antioxidant, lipid-lowering, gallbladder, and other biological activities. It has been applied in food, medicine, animal production, and other fields. Studies have shown that curcumin can reduce lipid levels, enhance anti-inflammatory and antioxidant capacities, activate IR signaling pathways, considerably prevent blood sugar rise in mice, reduce IR, and improve insulin sensitivity (Chen, 2022). Furthermore, curcumin can reduce ferroptosis and associated myocardial damage in diabetic mice via the NRF2-glutathione peroxidase 4 (GPx4)/heme oxygenase-1 (HO-1) pathway (Wei Z. et al., 2022). Curcumin analogs can also inhibit the NF-κB signaling pathway, myocardial inflammation, and improve diabetic cardiomyopathy (Wang M. et al., 2022). Moreover, curcumin inhibits the glycosylation of myocardial proteins in mice induced by a high-fructose diet (Leon-Garcia et al., 2022), improves the cardiomyopathy of diabetic rats induced by STZ combined with a high-fat and high-sugar diet, and activates the silencing information regulator 1(Sirt1)-fork head transcription factor (FoxO1) and PI3K-AKT pathways, alleviating myocardial oxidative stress and inhibiting apoptosis (Ren et al., 2020). C66, a derivative mentioned in the literature, has been shown to ameliorate obesity-related cardiomyopathy induced by a high-fat diet and palmitic acid-stimulated cardiomyocyte injury in H9c2 rats by inhibiting the JNK signaling pathway (Ye et al., 2021). The rose is the dried bud of the Rosaceae family, which has high ornamental, edible, and medicinal value. *Rosa rugosa* Thunb. polyphenol extract, a polyphenol-rich rose extract, inhibits the activity of α-glucosidase, an enzyme that breaks down complex carbohydrates into absorbable monosaccharides in the small intestine, improving glycogen metabolism and regulating the blood glucose of T2DM rats (Liu et al., 2017). Resveratrol attenuates diabetic cardiac microvascular injury through antioxidative stress (Cai, 2024).

Terpenoids represent the largest group of natural compounds. Volatile oils, resins, rubber, and carotenes are terpenoids, most of which have various physiological activities. Diterpenoids extracted from rock sugar grass can protect islets and alleviate IR (Sun et al., 2020). Euscaphic acid, Tormentic acid, Corosolic acid, Maslinic acid, Oleanolic acid, and Ursolic acid belong to terpenoids and can inhibit alpha glycosidic enzyme activity *in vitro* and inhibit the absorption of glucose (Chen J. W. et al., 2020). These findings suggest that it may offer promising avenues for treating DM and its related cardiovascular complications.

In addition, based on the principle that structure determines function, the derivatives of these compounds may be used for the prevention and treatment of diabetes and its cardiovascular complications. TPX, derived from the mangiferin derivative 1,3,6,7-tetraallylloxy ketone, can restore the insulin signaling pathway, increase liver glycogen synthesis, and protect against IR caused by glucose metabolism disorders in liver cells (Fan et al., 2023).

## 5 Discussion

**FIGURE 1:**
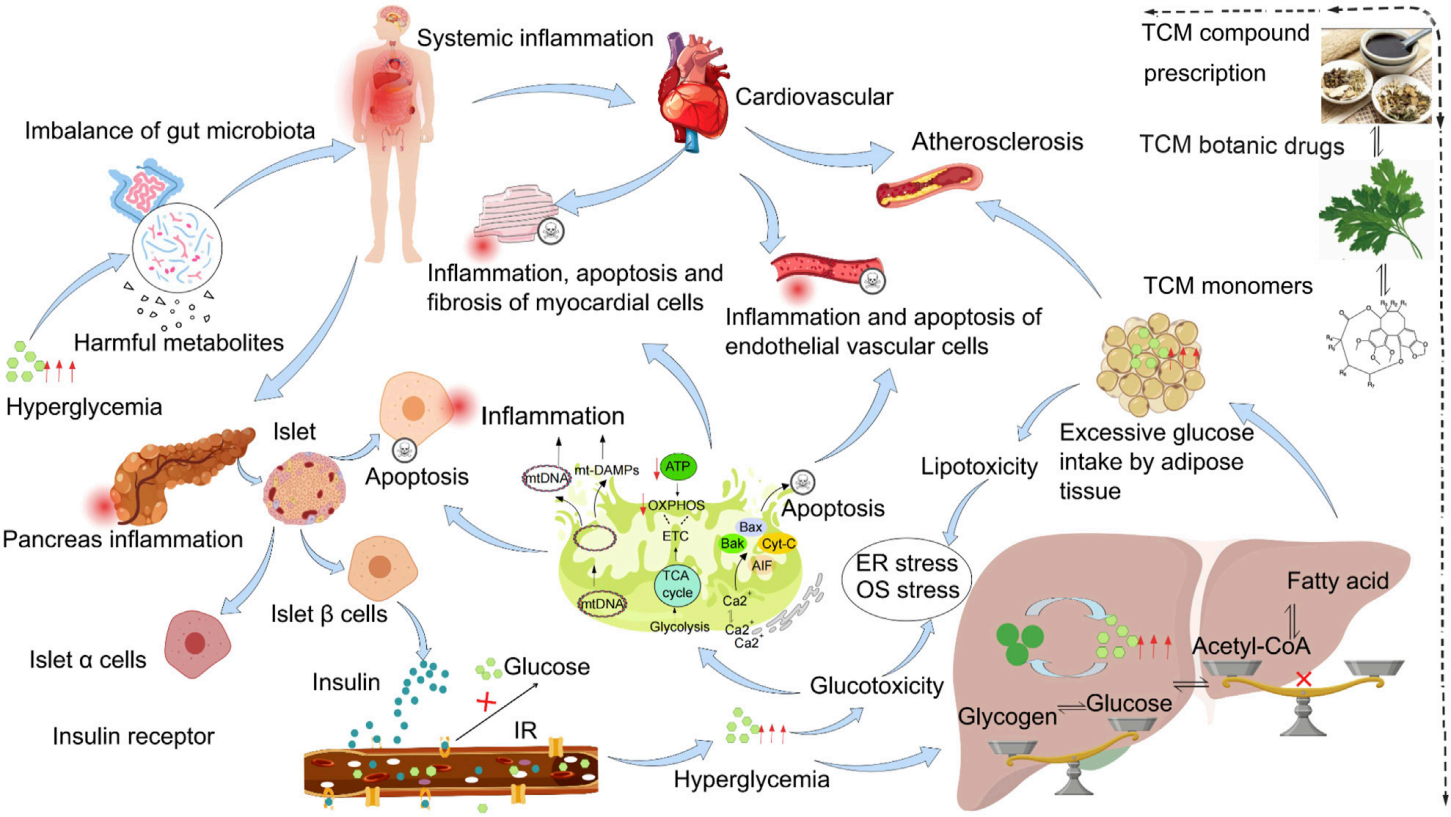
Schematic Diagram of the Pathogenesis of DACC. The pathogenesis of DACC involves multiple interconnected mechanisms, including insulin resistance, gut microbiota dysbiosis, oxidative stress, disorders of glucose and lipid metabolism, inflammation, and apoptosis. These factors interact synergistically, contributing to the initiation and progression of DACC. In the diagram, the red upward arrow (↑) indicates an elevation or increase in a specific factor. The red downward arrow (↓) signifies a reduction or decrease. The red “×” denotes an inhibitory effect. The blue curved arrow represents promotion or generation of a process. The black arrow illustrates the occurrence of a biochemical process. The red halo symbolizes inflammation, while the skull icon represents apoptosis. This schematic highlights the complex interplay of these mechanisms, providing a comprehensive overview of the multifactorial nature of DACC pathogenes.

The number of people diagnosed with DM is increasing (Collaborators, 2023). The risk of cardiovascular disease in people with DM is approximately 2.5 times that in people without DM, contributing to the impaired life expectancy of patients with DM (Bragg et al., 2017; Bragg et al., 2016). Although numerous standard biomedical drugs are currently being researched for the treatment of DM and related diseases. However, owing to the side effects and toxicity of these drugs (Table 2), it appears that the single use of standard biomedical treatment drugs is not the optimal plan for patients with chronic diseases who need to take them for a long time. As research into DACC advances, the preventive and therapeutic effects of TCM have been increasingly affirmed. TCM compound prescriptions, botanical drugs, and monomer components have been shown to have positive effects on DACC prevention and treatment. The pathogenesis of includes mainly multiple factors (such as intestinal flora disorders, inflammation, oxidative stress, ER stress, and metabolic disorders) induced by impaired islet function, insufficient insulin secretion, or a decreased ability to perceive glucose (Figure 1). Long-term high-sugar stimulation causes inflammation and damages the normal physiological functions of vascular endothelial cells and cardiomyocytes. In addition, high sugar levels cause oxidative stress in the myocardium and cardiovascular cells, resulting in cardiovascular damage. Hyperglycemia stimulates the proliferation of vascular smooth muscle cells and collagen synthesis, leading to fibrosis and hardening of the vascular wall, causing blood vessels to lose their elasticity, increasing vascular resistance and pressure, and inducing diabetic cardiovascular disease. Long-term high sugar consumption stimulates intestinal flora disorder, which can destroy the normal metabolism of the body, enhance the oxidative stress response in the body, and through the secretion of harmful bacterial endotoxins, shift to cardiovascular circulation and aggravate cardiovascular system inflammation. This review explores the relevant mechanisms of TCM compound prescriptions, TCM botanical drugs, and active compounds in the prevention and treatment of DACC in recent years. Also, it provides the necessary theoretical basis for the modern development of TCM. An increasing number of studies have suggested that the effect of integrated Chinese and standard biomedical treatments on the treatment of DM yields better outcomes than standard biomedical treatments alone (Gu et al., 2018; Ma K. et al., 2023; Zhao et al., 2019). Studies have shown that Liuwei Dihuang Pill (decoction) combined with metformin for the treatment of T2DM may reduce the adverse effects of metformin (Zhao et al., 2019). Gu Yuming (Gu et al., 2018) reported that the common adverse events in the TCM group were gastrointestinal symptoms (nausea/vomiting, bloating, and diarrhea), nervous system symptoms, and hypoglycemia. However, no significant abnormalities in blood, liver, or kidney functions were observed in any of these studies. This may provide ideas for promoting combined treatments for DACC. Therefore, the use of TCM, either independently or in combination with standard biomedical therapies, represents a viable alternative for the prevention and treatment of DACC.

TCM has demonstrated unique advantages in the prevention and treatment of DACC, offering novel therapeutic approaches through its multicomponent, multitarget mechanisms of action. However, the clinical application of TCM for the management of DACC poses substantial challenges. First, the complexity of TCM components and issues related to standardization remain major hurdles. Each TCM botanical drug is a complex mixture of natural compounds that often contain various chemical constituents such as alkaloids, flavonoids, terpenoids, and polysaccharides. For instance, *ginseng* alpha wood contains multiple ginsenosides as well as volatile oils, polysaccharides, and amino acids, which interact synergistically. In compound formulations, the chemical complexity is further amplified, making it difficult to precisely elucidate pharmacological mechanisms. This complexity poses a challenge for quality control and drug development. For instance, when studying the mechanisms of action of a TCM formulation for DM treatment, it is challenging to identify the specific components that exert hypoglycemic effects and how they interact. Standardization issues also hinder the clinical application of TCM. The quality of TCM botanic materials varies considerably due to differences in geographical origin, cultivation environment, harvesting season, and processing methods. For instance, Angelica sinensis from Min County, Gansu Province, China, differs in its active component content from those in other regions. Even within the same region, variations in the climate and other factors can lead to inconsistent quality. In addition, the lack of uniform and precise standards in TCM processing further complicates quality control, because different processing techniques can yield products with varying efficacies. In the process of TCM preparations, the absence of standard quality control systems results in substantial variability in the composition and efficacy of products from different manufacturers, which undermines the accuracy and effectiveness for clinical applications. Second, the safety and adverse effects of TCM cannot be overlooked. Although TCM is often perceived to be natural and safe, this is not always the case. Some TCM herbs contain toxic components such as aconitine in Aconitum species, which can cause severe poisoning if improperly processed or overdosed. Moreover, the adverse effects of TCM may be insidious and delayed, making prompt detection difficult. Hepatotoxicity and nephrotoxicity were the most common adverse effects. For example, long-term use of certain compound formulations containing *Astragalus* L., *Salvia miltiorrhiza* Bunge, and *Coptis* Salisb. Is associated with liver injury. A case study reported that a patient with diabetes developed abnormal liver function 6 months after using a TCM formulation, which resolved after discontinuation (Pan et al., 2020). Similarly, herbs such as *Polygonum multiflorum* and *Tripterygium wilfordii* have been linked to hepatotoxicity, potentially causing irreversible liver damage (Song J. et al., 2023). Mechanistically, certain TCM components such as anthraquinones and alkaloids may induce oxidative stress or directly damage hepatocytes. Chronic use of herbs containing aristolochic acid, such as *Aristolochia manshuriensis* Kom. and *Aristolochia fangchi* Y. C. Wu ex L. D. Chow and S. M. Hwang has been associated with nephrotoxicity, potentially leading to chronic renal failure (Omer Mohamed et al., 2020). Aristolochic acid may induce apoptosis and fibrosis in renal tubular epithelial cells. In addition, the combination of TCM with standard biomedical treatments, owing to their complex composition, may lead to drug interactions and increase the risk of adverse effects. For instance, the concurrent use of hypoglycemic herbs, such as Ginseng Alpha Wood and *Lycium barbarum* Mill. With standard biomedical treatments, antidiabetic drugs may increase the risk of severe hypoglycemia (Ni et al., 2024). This may be attributed to active components such as ginsenosides, which enhance insulin sensitivity or promote glucose utilization. However, current research on TCM safety remains limited and lacks a comprehensive safety evaluation system, making it difficult for clinicians and patients to accurately assess and manage potential risks. Furthermore, most existing studies have been limited to animal experiments or small-scale clinical trials, with a lack of large-scale, high-quality, multicenter, randomized controlled trials (RCTs). Future research should focus on elucidating the mechanisms of TCM, conducting high-quality multicenter RCTs, and investigating the synergistic effects and potential risks of combining TCM with standard biomedical treatments to advance the modernization and global recognition of TCM. Modern scientific techniques, such as network pharmacology and metabolomics, should be employed to investigate the active components and multi-target mechanisms of TCM to provide safer and more effective therapeutic options for DACC.

Despite the systematic review of TCM applications in DACC presented in this study, several limitations persist. Mechanistic studies addressing anti-inflammatory and gut microbiota regulation lack depth in molecular biology and cellular signaling pathways, making it difficult to precisely delineate the synergistic actions of complex components. Clinically, the scarcity of high-quality, large-sample, multicenter RCTs limits the reliability and generalizability of these findings. In addition, there is a notably deficiency in studies investigating the combined use of TCM with standard biomedical treatments, failing to provide adequate guidance for clinical practice. Otherwise, the methodological rigor of the studies incorporated in this review calls for careful and critical examination. In the realm of study design standardization, there seem to be several prevalent concerns. A significant number of studies have not adopted standardized approaches for estimating sample sizes. This lack of standardization can potentially lead to underpowered studies, which may in turn impact the general applicability of the findings, particularly when attempting to discern differences in treatment effects across various patient subgroups. Furthermore, the documentation of randomization protocols appears to be somewhat insufficient, and the setup of control groups could be optimized. When assessing the interventions of TCM in the context of DACC, the potential synergistic or antagonistic interactions between conventional therapies and TCM botanical drugs formulations have yet to be comprehensively evaluated. In addition, the over - reliance on subjective patient - reported outcomes raises some questions regarding data reliability. Since these outcomes are susceptible to recall bias and placebo effects, they might not provide the most robust basis for drawing firm conclusions. As for traditional pre - clinical models, there are certain aspects that could be improved. Rodent models exhibit species - specific variations in glucose homeostasis mechanisms and cardiovascular drug metabolism pathways, and traditional in - vitro islet cell models struggle to fully replicate the intricate paracrine signaling networks and three - dimensional niche architecture characteristic of the human pancreatic microenvironment. These gaps require further investigation.

Future research on the use of TCM for the prevention and treatment of DACC is both challenging and promising. Modern separation technologies, such as chromatography-mass spectrometry, should be employed to isolate and identify active TCM components and elucidate their mechanisms of action to establish a solid theoretical foundation for clinical applications. However, large-scale, high-quality, multicenter RCTs are urgently required to validate the efficacy and safety of TCM. Exploring the synergistic effects and potential risks of combining TCM with standard biomedical treatments will enable the development of scientifically sound treatment protocols and offer patients better therapeutic options. Future research should adopt prospective designs and employ validated methods to ensure high - level effectiveness of the studies. Furthermore, leveraging modern scientific techniques, such as network pharmacology and metabolomics, will drive the modernization of TCM and enhance its global recognition and application. This will enable TCM to play a pivotal role in the global prevention and treatment of diabetes, thereby contributing substantially to this field.

In summary, TCM has made remarkable progress in the prevention and treatment of DACC. With the continuous development of biomedicine, science, and technology, the mechanism of action of TCM in the treatment of DACC is expected to improve. More advanced modern bioscience technologies are expected to help researchers identify targets and apply TCM with multiple components, mechanisms, and targets for the prevention and treatment of DACC. In addition, research on the combination of TCM and standard biomedical treatments in the treatment of DACC will be continuously deepened, and basic research and clinical trials of TCM will also achieve a comprehensive transformation. New related technologies may be the primary tool for overcoming the problems associated with the prevention and treatment of various diseases, further promoting the application of TCM in clinical settings.
